# Injectable Platelet-Rich Fibrin (I-PRF) Administered to Temporomandibular Joint Cavities: A Scoping Review

**DOI:** 10.3390/jcm12093326

**Published:** 2023-05-07

**Authors:** Marcin Sielski, Kamila Chęcińska, Maciej Chęciński, Maciej Sikora

**Affiliations:** 1 Department of Maxillofacial Surgery, Hospital of the Ministry of Interior, Wojska Polskiego 51, 25-375 Kielce, Poland; 2 Department of Glass Technology and Amorphous Coatings, Faculty of Materials Science and Ceramics, AGH University of Science and Technology, Mickiewicza 30, 30-059 Kraków, Poland; 3 Department of Oral Surgery, Preventive Medicine Center, Komorowskiego 12, 30-106 Kraków, Poland; maciej@checinscy.pl; 4 Department of Biochemistry and Medical Chemistry, Pomeranian Medical University, Powstańców Wielkopolskich 72, 70-111 Szczecin, Poland

**Keywords:** temporomandibular joint, temporomandibular disorders, intra-articular injections, platelet-rich fibrin, arthrocentesis

## Abstract

The aim of this review was to systematically map the research on the intra-articular administration of injectable platelet-rich fibrin (I-PRF) to the temporomandibular joints (TMJs). Medical databases covered by the ACM, BASE, Google, NLM, and ResearchGate were searched on 23 February 2023. The assessment of the level of evidence was based on the Oxford Center for Evidence-Based Medicine 2011 scale. The risk of bias was assessed for randomized controlled trials with the RoB2 tool. Extracted data were tabulated, and the changes in effect values were calculated. A total of eight studies qualified, of which five trials on 213 patients were randomized and controlled (RCTs). In each of the RTC study groups, arthrocentesis was performed, and 1–2 mL per joint of I-PRF (700 rpm/3 min/60 g centrifugation) was administered. Articular pain in three months decreased to 0–25% of the initial pre-interventional values in the study and 38–50% in the control groups. Mandible mobility increased to 121–153% and 115–120% in the I-PRF groups and controls, respectively. The main limitations of the evidence were the small number of RCTs and the lack of any RCT study groups receiving I-PRF without prior arthrocentesis. In conclusion, supplementing the temporomandibular joint rinsing with I-PRF administration further relieves pain and improves mandible mobility. The lack of RCTs on the intra-articular administration of I-PRF as a stand-alone procedure encourages further research. This research received no external funding. The review protocol has not been previously published.

## 1. Introduction

### 1.1. Rationale

Temporomandibular disorders (TMDs) are a group of disorders affecting the temporomandibular joint (TMJ) and the muscles that control its movements [1,2,3]. Typical symptoms of TMD are pain or tenderness in the preauricular area, difficulty opening or closing the mouth, and a clicking sensation within the TMJs [1,4,5,6]. Treatment of TMD, depending on the etiology, may include pharmacotherapy, physiotherapy, splint therapy, minimally invasive surgical procedures such as intramuscular and joint punctures, arthroscopy, and in some cases, open surgery [7,8,9,10,11,12,13]. Among the commonly used methods based on punctures into the TMJs cavities, there are pumping arthrocentesis, two-way lavage, and intra-articular administration of various substances [14,15,16,17]. The injectables studied so far include corticosteroids (CS), hyaluronic acid (HA), hypertonic dextrose (HD), analgesics, ozone, and auto-derived preparations [5,15,18,19,20,21,22]. The currently used autografts are mainly substances obtained from the patient’s centrifuged blood: platelet-rich plasma (PRP), plasma rich in growth factor (PRGF), and injectable platelet-rich fibrin (I-PRF), of the most favorable composition [21,22,23,24,25,26].

Arthrocentesis with infusion fluids and intra-articular administration of popularly used injectables (CS, HA) generally improves mandibular mobility, reduces the intensity of articular acoustic symptoms and relieves pain, but does not lead to the regeneration of bone and cartilage erosions [14,15,16,27]. Therefore, the search for an effective method of treating less advanced forms of degenerative joint disease is highly justified [28]. Such therapy could shift the indications for arthroscopy and open surgery of TMJs towards more severe morphological disorders [22,29,30]. The latest clinical research sees the most promising solution in the transplantation of autologous mesenchymal stem cells (MSCs), primarily from adipose tissue [23,31,32,33]. Unfavorably, the implantation of fat preparations carries the risk of iatrogenic embolism, which can even lead to vision loss [34,35,36]. Therefore, stimulation of the stem cell proliferation and differentiation with the use of an appropriate growth factor concentrate, i.e., I-PRF, seems to be safer, technically easier, faster, and cheaper than adipose MSCs autografting [33,37].

Therefore, it seems that I-PRF is currently the most appropriate injectable in the treatment of TMDs. However, the research on intra-articularly administered I-PRF has not been systematically reviewed so far.

### 1.2. Objectives

The purpose of this review is to systematically map available data on the effectiveness of I-PRF administration in the TMJ cavities in the treatment of TMDs.

## 2. Methods

Subsequent stages of the review were carried out in accordance with the Preferred Reporting Items for Systematic Reviews and Meta-Analyses (PRISMA) protocols [38,39].

### 2.1. Eligibility Criteria

The criteria for inclusion and exclusion of studies were defined in accordance with the Patients, Intervention, Control, Outcomes (PICO) framework (Table 1) [40]. Only primary clinical studies were allowed. Diagnoses were not limited to specific disease entities. For quantitative purposes, any comparison methods based on intra-articular injections were allowed. Ranges of articular pain, mobility in the joint, and any TMD severity scales were accepted as eligible quantitative outcomes. There were no time limits for the publication dates of the reports.

### 2.2. Information Sources and Search Strategy

The following engines were used to search medical databases for papers of all types, including articles, conference papers, and ongoing trial reports, regardless of study design: Guide to Computing Literature (ACM), Bielefeld Academic Search Engine (BASE), Google Scholar (GS), National Library of Medicine: ClinicalTrials.gov (NLM-CT), National Library of Medicine: PubMed (NLM-PM), and ResearchGate (RG). All searches were made on 23 February 2023. The following search strategy was used: “(i-prf OR “injectable platelet-rich fibrin”) AND temporomandibular”. In order to prevent the Google search engine from deviating from the content of the query, the “allintitle” (GS-AT) and “Sort by date” (GS-SD) commands were used. Detailed queries tailored to the specifics of individual search engines are listed in Table 2.

### 2.3. Selection Process

All records were entered into the Rayyan (Cambridge, MA, USA) automation tool and subjected to manual deduplication by two authors (K.C. and M.C.) [41]. In the next stage, the same authors performed a blind screening of titles and abstracts in accordance with the PICO criteria described above [40]. The agreement of assessments was expressed by Cohen’s kappa coefficient [42]. Records identified unanimously as ineligible have been removed. The remaining elements were transferred to the full-text analysis phase conducted independently by two researchers (M.Sie. and M.C.). In case of discrepancies regarding eligibility, the third judge (K.C.) made the final decision.

### 2.4. Data Collection Process

Data identifying individual studies, characteristics of study groups, and results in eligible domains were extracted from the content of articles by two independent researchers (M.Sie. and M.C.). In cases of inaccuracies, arrangements were made by consensus. Data was obtained only from the published content of articles and supplementary materials. No automation tools were used at this stage.

### 2.5. Data Items

For the purposes of the characteristics of the study groups, the first author of the report and the year of publication, the total number of patients, diagnosis, centrifugation protocol, study groups, number of patients in each study group, one-time amount of I-PRF administered (dose), number of administrations in the therapy protocol (number of doses), and substances administered in control groups (comparators) were collected. In the absence of data, this fact was noted in the summary table.

Each separate group of patients administered I-PRF was treated as a separate study group. Each of the groups that received a different substance (including placebo or lavage fluid) was treated as a control. In the domain of articular pain, values were taken from the visual analog scale (VAS) by default, and in the absence of this data, a numeric rating scale (NRS) or any other pain rating scale [43]. In the case of pain assessments in various situations, the one closest to physiological mobility, i.e., chewing, was selected. For the assessment of mandibular mobility, the values of the maximum unassisted opening of the mouth were extracted most willingly (regardless of the reference points) [43,44]. In the absence of these values, maximal painless opening, maximal manual assisted opening or lateral movements were selected, respectively [43,44]. In the case of the presence of TMDs staging scores according to different scales (e.g., Oral Health Impact Profile—Temporomandibular Disorders, Eight-item Jaw Function Limitations Scale, Helkimo Index, Fonseca Questionnaire, etc.), the values of all of them were collected [43,45].

### 2.6. Study Risk of Bias Assessment

All source studies levels of evidence were assessed in terms of treatment benefits according to Oxford Centre for Evidence-Based Medicine 2011 scale [46]. The risk of bias in controlled studies was determined using the revised Cochrane risk-of-bias tool for randomized trials (RoB2) [47]. The assessment was made by two authors (M.Sie. and M.C.) without the use of automation tools. Randomized controlled trials with no high risk of bias in any domain of the RoB2 tool were processed further [47].

### 2.7. Synthesis Methods

Quantitative eligibility was assessed by evaluating the completeness of data in at least one outcomes domain for each of the study groups. Effect variables in pain relief, increasing mandibular mobility, and reducing the value of dysfunction indices have been made independent of the specificity of the protocols of individual studies by calculating relative values according to the formula:

e = f/i × 100%,

where e is the effect, f is the final value of the variable, and i is the initial value of the variable. For pain, values below 100% (baseline value) indicate treatment relief. An increase in the mandibular opening range above 100% proves intervention effectiveness in improving mandible mobility. The decrease in the TMDs severity indices below 100% demonstrates the beneficial effect of the therapy on the overall assessment of the TMJs function.

## 3. Results

### 3.1. Study Selection

**Figure 1:**
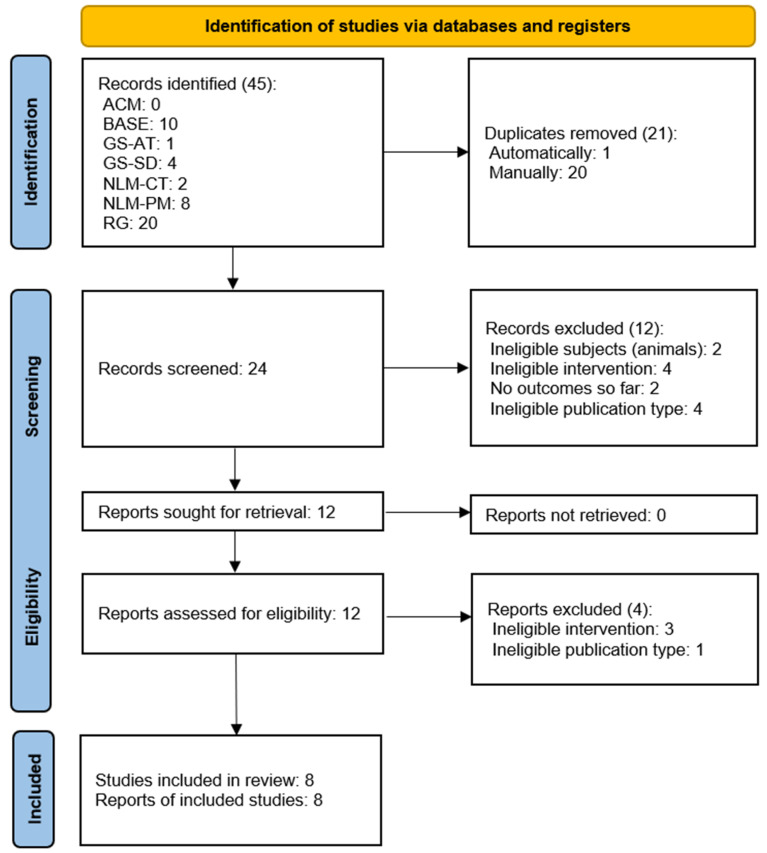
PRISMA flow diagram.

Of the 45 records identified, eight studies were ultimately included in the review. The entire selection process is illustrated in Figure 1. The number of records found using each search engine is presented in Table 3. The agreement of the judges’ decisions at the screening stage was Cohen’s k = 0.83, which means almost perfect agreement. Items that were in dispute were moved to the full-text analysis stage in order to reach an agreement.

At the stage of full-text eligibility, four studies were rejected in accordance with the above PICO criteria [48,49,50,51]. The detailed reasons for these decisions are presented in Table 4.

### 3.2. Study Characteristics

Table 5 presents the basic data characterizing the study groups in the context of the design of the included trials [26,52,53,54,55,56,57,58].

The number of patients in the study groups of controlled studies ranged from 18 to 24. The diagnoses each time fell within the canon of typical indications for injection therapy but were inhomogeneous through the studies. Every I-PRF administration in controlled trials was preceded by arthrocentesis. Due to the centrifuge settings being consistent for all studies to obtain I-PRF, these values have been omitted from the table. Each centrifugation protocol was 700× *g* rpm for 3 min with a relative force of 60 g. The volume of the injected blood preparation resulted directly from the volume of the upper joint cavity and ranged from 1 to 2 mL [59]. The puncture protocol each time suggested administration to the upper TMJ compartment. However, the lack of imaging control does not allow us to be sure of the precise place of deposition [59]. The number of doses of the preparation used ranged from one to four, and in the case of multi-dosing, the intervals were seven days in controlled trials. The study intervention was compared each time with arthrocentesis and in two of the trials, additionally also, with arthrocentesis with HA administration.

### 3.3. Risk of Bias in Studies

The evaluation of the research based on data from the reports showed that three out of five analyzed controlled studies met the minimum requirements for qualification as randomized controlled trials with a moderate risk of bias (Table 6) [53,54,55]. The other two papers reported a retrospective analysis of medical records, which prevented their inclusion in the synthesis of the results [56,57].

### 3.4. Results of Individual Studies

The results of individual randomized controlled studies are presented below [53,54,55]. This section omitted studies discussed in qualitative terms only.

#### 3.4.1. Articular Pain

**Figure 2:**
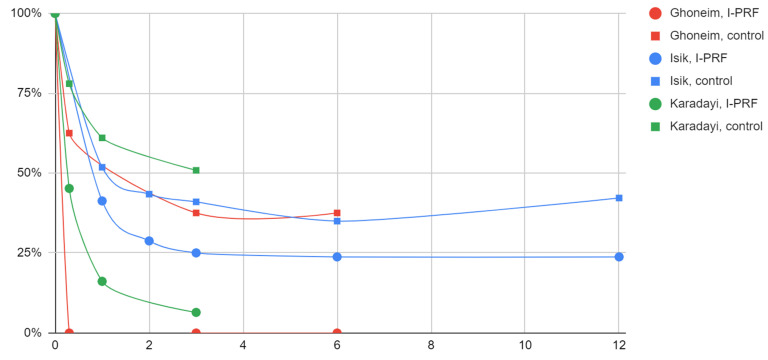
Effect of arthrocentesis with I-PRF versus arthrocentesis alone on articular pain over time (months).

In all study groups (arthrocentesis and I-PRF administration) and control groups (arthrocentesis) after the intervention, articular pain values significantly decreased compared to the values before treatment (Table 7, Figure 2). After three months of treatment, pain intensity ranged from 0% to 25% of baseline values in the study groups and from 38 to 50% in the control groups. Further follow-up showed no improvement, and in the only control group observed for more than six months, it brought a slight recurrence of symptoms, while the therapeutic effect was maintained in the parallel I-PRF group.

#### 3.4.2. Mandibular Mobility

**Figure 3:**
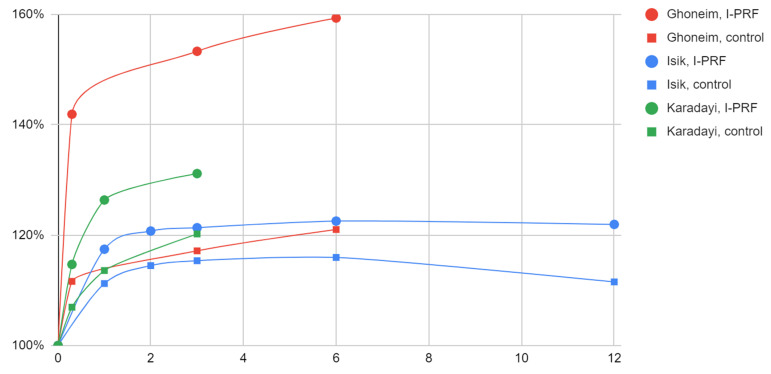
Effect of arthrocentesis with I-PRF versus arthrocentesis alone on mandibular mobility over time (months).

Intra-articular injections improved the mobility of the mandible in each of the discussed groups of patients (Table 8, Figure 3). Each time the effect was better in the study groups (from 121% to 153% of the initial mandibular abduction) than in the control groups (from 115% to 120%). Significant improvement was observed already after seven to ten days, but approximately maximum values were observed in the period of two to twelve months after the first intervention. In the observation longer than six months, a decrease of more than a millimeter in the value of mouth opening was observed in the control group but not in the study group.

#### 3.4.3. TMD Indices

Other scales of TMDs intensity used were the assessment of the presence of acoustic symptoms from TMJs and the Helkimo index (Table 9). Complete resolution of the clicking has been demonstrated after arthrocentesis and a single administration of I-PRF, but not after arthrocentesis alone. The Helkimo index values after three months reached a discrepancy from 13% of the initial value for the study group to 41% for the control group.

### 3.5. Other Studies

In a study by Muhammad et al., a decrease in the intensity of articular pain was observed in the group receiving intra-articular I-PRF, the group treated with ultrasound, and the group receiving a combination of both therapeutic methods [58]. There was no statistically significant difference between the groups, which, according to the authors, negates the synergistic activity between the two treatments while proving the analgesic efficacy of both [58].

In an uncontrolled study by Albilia et al., intracavitary administration of I-PRF was effective in alleviating pain [26]. The study design allowed for the continuation of therapy only in respondents, which resulted in identifying a group of about 30% of patients who did not improve in the course of the discussed treatment [26]. A higher percentage of improving patients correlated with a higher stage in the Wilkes classification [26].

Manafikhi et al. observed patients for the presence of acoustic symptoms and achieved 100% effectiveness in relieving them for a period of six months after two administrations of I-PRF [52]. Due to the preliminary nature of this prospective study, no control group was planned, which did not allow to prove that the type of substance had an impact on the treatment effect [52].

## 4. Discussion

### 4.1. General Interpretation of the Results

Additional administration of I-PRF to the TMJs cavities as a complement to arthrocentesis is more effective than arthrocentesis alone in each of the examined domains [53,54,55,56,57]. However, there are no studies that would allow the assessment of intra-articular administration of I-PRF as a stand-alone procedure.

#### 4.1.1. Arthrocentesis with I-PRF Administration versus Sole Arthrocentesis

This review demonstrated that after three months of observation, the effect of combined therapy (arthrocentesis with I-PRF) is superior to the procedure without I-PRF by 16% to 43% in relieving articular pain and from 6% to 36% in increasing the range of mandibular abduction [53,54,55]. The smallest discrepancies were observed in a study with four interventions [54]. A comparison of the results from various reports for the study groups alone showed that the therapeutic effect in both domains is better with a single arthrocentesis with I-PRF administration than in the case of a four-fold repetition of the intervention [53,54,55]. Differences between the results of the control groups across the studies were not as pronounced [53,54,55].

#### 4.1.2. Arthrocentesis with I-PRF Administration versus Arthrocentesis with HA Administration

The retrospective nature of studies with control groups of patients treated with arthrocentesis combined with the administration of HA did not allow their results to be included in the quantitative assessment [56,57]. Data presented by Torul et al. demonstrates the effectiveness of arthrocentesis with HA injection at a level similar to arthrocentesis alone in terms of both articular pain and the extent of mouth opening [56]. In opposition to them was the administration of I-PRF after lavage, which in both domains gave clearly better results [56]. In the report by Yuce et al., rinsing the TMJ preceded with I-PRF or HA injection brought a similar effect up to six months after the intervention, clearly superior to arthrocentesis alone [57]. In the course of further follow-up, there was a pronounced advantage of I-PRF over HA [57]. These results were consistent for both pain and abduction domains [57].

### 4.2. Limitations of the Evidence

The lack of concealment of I-PRF administration from the patients, common for the analyzed randomized controlled studies, was not classified as increasing the risk of bias to a “High” value [53,54,55]. This decision was motivated by the fact that blinding the intervention from the patient would require redundant blood collection in control groups. Thus none of the studies included in the quantitative analysis were assessed as having a high risk of bias [53,54,55]. Another relatively easily eliminable problem of the lack of blinding of groups of patients from researchers making measurements in the course of post-intervention follow-up was unnecessarily present [53,54,55].

The overall small number of studies considering intracavitary administration of I-PRF as part of TMD therapy makes it difficult to assess the efficiency of this preparation [26,48,49,52,53,54,55,56,57,58]. The few studies comparing arthrocentesis and I-PRF with any other injection therapy were included in the quantitative section of this systematic review [53,54,55]. However, the absence of any randomized controlled trials examining the administration of I-PRF as a stand-alone further exacerbates the problem of evidence limitation.

### 4.3. Limitations of the Review Processes

In the course of the review, only English-language search queries were used, which limited the results to those containing at least the title in English. Although searches have been carried out using a range of engines covering numerous medical databases, the completeness of the search results cannot be guaranteed.

### 4.4. Rejected Reports

The overall availability of research on the administration of I-PRF into TMJs cavities is so low that it seems justified to briefly mention reports rejected in this review [26,48,49,50,51,52,58,60,61]. Two clinical trials are currently reported as ongoing, but their results have not yet been published [60,61]. Another three trials with additional interventions were rejected during the selection process [48,49,51]. In the first of these trials, patients underwent additional conservative treatment (splint therapy and physiotherapy) [49]. Due to the complexity of the therapy, the assessment of the I-PRF component was not possible [49]. The study showed similar effects of the intracavitary administrations regardless of the injectable used: I-PRF, PRP, or HA [49]. In the second of the ineligible intervention papers, apart from intra-articular injections, pericapsular injections and immobilization were used, which enabled effective treatment of TMJ dislocations [48]. A reduction in maximal mouth opening was achieved, despite relying on intra-articular I-PRF, the same intervention that other investigators use to increase mandibular mobility [48]. The third of the discussed reports is a retrospective evaluation of a case series in which, as a result of combining arthroscopy with intra-articular administration of liquid PRF, a decrease in the severity of articular pain by about six points on the VAS scale and an increase of more than 40% in mandibular mobility was observed during an eight-month follow-up [51]. The lack of a control group makes it impossible to assess the impact of the administration of the blood product on the complex treatment results [51].

### 4.5. Future Research

Temporomandibular joint arthropuncture is gradually becoming common due to its relatively easy technique [62,63,64]. Controversies over the improvement of the procedure do not detract from the proven effectiveness of arthrocentesis and intra-articular injections, even when performed blindly [59,62,63]. The most proven is the reduction of articular pain and the increase in the range of motion of the mandible [5,15]. This can be achieved either by arthrocentesis or by administering approximately 1–2 mL of injectable per side [59]. Due to the smaller volume, it can be done with a single puncture and without the need for multiple withdrawals and replenishment of fluid in the joint cavity [14,15,16,22,30]. The simplification of the procedure in relation to known arthrocentesis techniques (double-needle, two-way needle, pumping) obviously reduces the risk of complications and the duration of the procedure. It can be presumed that it also increases the patient’s comfort, thus, the acceptance of the intervention. In the technique of single-needle, single-puncture administration into the TMJ cavity, CS, HA and PRP have been used primarily [5,24,65,66,67,68]. Due to the discussed advantages of I-PRF over the above-mentioned substances, it seems reasonable to evaluate the effects of I-PRF injections not preceded by arthrocentesis. The results of this meta-analysis suggest the possibility of a beneficial effect of the administration of I-PRF alone on the functioning of the TMJ, and thus the reduction of articular pain, mandible abduction restriction and acoustic symptoms severity. Studies comparing the results of I-PRF treatment (with and without arthrocentesis) in the context of the regenerative capacity of this preparation on TMJ structures would also be desirable.

## 5. Conclusions

Complementing arthrocentesis of the temporomandibular joint with injectable platelet-rich fibrin further reduces articular pain and increases mandible mobility. The lack of studies on the intra-articular administration of injectable platelet-rich fibrin as a stand-alone procedure encourages further research.

## Figures and Tables

**Table 1:** Eligibility criteria.

Domain	Inclusion Criteria	Exclusion Criteria
Patients	Diagnosis of TMDs in human patients	TMDs as a symptom of generalized joint disease
Intervention	I-PRF intra-articular injection alone or preceded by arthrocentesis	Additional interventions
Control	One of the following: (1) arthrocentesis; (2) placebo injection; (3) hyaluronic acid administration	Not applicable
Outcomes	At least one of the following: (1) TMJ articular pain; (2) mandibular mobility; (3) TMD severity scale	Values of variables expressed qualitatively (present/absent) without using any scale

**Table 2:** Search strategy variants.

Search Engine	Query
ACM	[[All: i-prf] OR [All: “injectable platelet-rich fibrin”]] AND [All: temporomandibular]
BASE	(i-prf OR “injectable platelet-rich fibrin”) AND temporomandibular
GS-AT	allintitle: (i-prf OR “injectable platelet-rich fibrin”) AND temporomandibular
GS-SD	(i-prf OR “injectable platelet-rich fibrin”) AND temporomandibular; “Sort by date” filter
NLM-CT	(i-prf OR “injectable platelet-rich fibrin”) AND temporomandibular
NLM-PM	(i-prf OR “injectable platelet-rich fibrin”) AND temporomandibular
RG	(“i-prf” OR “injectable platelet-rich fibrin”) AND “temporomandibular”

**Table 3:** Search results.

Search Engine	Number of Records
ACM	0
BASE	10
GS-AT	1
GS-SD	4
NLM-CT	2
NLM-PM	8
RG	20
Total:	45

**Table 4:** Studies rejected at the full-text evaluation stage.

First Author, Publication Year	Title	Reason for Rejection
Vingender, 2023 [49]	Evaluation of the efficiency of hyaluronic acid, PRP and I-PRF intra-articular injections in the treatment of internal derangement of the temporomandibular joint: A prospective study.	Additional interventions (splint therapy, physiotherapy)
da Silva Lima, 2022 [50]	Ácido Hialurônico Vs Fibrina Rica Em Plaquetas: Qualutilizar Em Tratamentos De Osteoartritetemporomandibular?	Review paper
Gonzalez, 2021 [51]	Clinical outcomes of operative arthroscopy and temporomandibular medical infiltration with platelet-rich fibrin in upper and lower articular space	Additional interventions (arthroscopy)
Baiomy, 2019 [48]	Versatility of Injectable Platelet Rich Fibrin versus autologous blood injection in the Treatment of Chronic Recurrent Temporomandibular Joint Dislocation	Additional interventions (pericapsular injection, immobilization)

**Table 5:** Characteristics of the study groups.

First Author, Publication Year	Total Number of Patients/Patients in the I-PRF Group	Diagnosis	Dose	Number of Doses/Interval	Comparators	Level of Evidence
Ghoneim, 2022 [53]	40/20	Disc displacement with reduction	1.5 mL intra-articularly	1/N/A	AC	2
Isik, 2022 [54]	36/18	TMJ osteoarthritis	1 mL intra-articularly	4/1 week	AC	2
Manafikhi,2022 [52]	20/20	Unilateral TMJ acoustic symptoms	1 mL intra-articularly	2/1 week	None	4
Muhammad, 2022 [58]	30/10	TMDs	1.5 mL intra-articularly	1/N/A	Ineligible (ultra-sound therapy)	3
Karadayi, 2021 [55]	36/18	Unilateral TMD	maximum of 2 mL intra-articularly	1/N/A	AC	2
Torul, 2021 [56]	54/18	TMJ internal derangement(Wilkes stage III)	1 mL intra-articularly	1/N/A	AC; AC + HA injection	3
Albilia, 2020 [26]	37/37	TMJ internal derangement(Wilkes stage I–V)	1.5–2 mL intra-articularly	Various/2 weeks	None	4
Yuce, 2020 [57]	47/24	TMJ internal derangement	2 mL intra-articularly	3/1 week	AC; AC + HA injection	3

TMJ—temporomandibular joint; I-PRF—injectable platelet-rich fibrin; PRP—platelet-rich plasma; AC—arthrocentesis; HA—hyaluronic acid.

**Table 6:** Risk of bias assessment summary.

First Author, Publication Year	Study Design	Randomization Process	Deviations from the Intended Interventions	Missing Outcome Data	Measurement of the Outcome	Selection of the Reported Result	Overall Risk of Bias
Ghoneim, 2022 [53]	Randomized controlled trial	Some concerns	Some concerns	Low	Some concerns	Low	Some concerns
Isik, 2022 [54]	Randomized controlled trial	Low	Some concerns	Low	Some concerns	Low	Some concerns
Karadayi, 2021 [55]	Randomized controlled trial	Low	Some concerns	Some concerns	Some concerns	Low	Some concerns
Torul, 2021 [56]	Retrospective study	N/A	N/A	N/A	N/A	N/A	N/A
Yuce, 2020 [57]	Retrospective study	N/A	N/A	N/A	N/A	N/A	N/A

N/A—not applicable.

**Table 7:** Articular pain in the study and control groups.

First Author, Publication Year	Patient Group (Number of Patients)	Initial Value	After 7–10 Days	After 1 Month	After 2 Months	After 3 Months	After 6 Months	After 12 Months	Effect after 3 Months (the Lower the Better)
Ghoneim, 2022 [53]	AC + I-PRF	6.0	0.0 *	N/A	N/A	0.0 *	0.0 *	N/A	0.0% *
	AC	8.0	5.0 *	N/A	N/A	3.0 *	3.0 *	N/A	37.5% *
Isik, 2022 [54]	AC + I-PRF	8.0	N/A	3.3 *	2.3 *	2.0 *	1.9 *	1.9 *	25.0% *
	AC	8.3	N/A	4.3 *	3.6 *	3.4 *	2.9 *	3.5 *	41.0% *
Karadayi, 2021 [55]	AC + I-PRF	6.2	2.8 *	1.0 *	N/A	0.4 *	N/A	N/A	6.5% *
	AC	5.9	4.6	3.6 *	N/A	3.0 *	N/A	N/A	50.1% *

I-PRF—injectable platelet-rich fibrin; AC—arthrocentesis; N/A—not applicable; * *p* < 0.05.

**Table 8:** Mandibular mobility in the study and control groups. Values in millimeters.

First Author, Publication Year	Patient Group	Initial Value	After 7–10 Days	After 1 Month	After 2 Months	After 3 Months	After 6 Months	After 12 Months	Effect after 3 Months (the Higher the Better)
Ghoneim, 2022 [53]	AC + I-PRF	31.5	44.7 *	N/A	N/A	48.3 *	50.2 *	N/A	153.3%
	AC	36.2	40.4 *	N/A	N/A	42.4 *	43.8 *	N/A	117.1% *
Isik, 2022 [54]	AC + I-PRF	33.3	N/A	39.1 *	40.2 *	40.4 *	40.8 *	40.6 *	121.3% *
	AC	33.9	N/A	37.7 *	38.8 *	39.1 *	39.3 *	37.8 *	115.3% *
Karadayi, 2021 [55]	AC + I-PRF	33.4	38.3	42.2 *	N/A	43.8 *	N/A	N/A	131.1% *
	AC	31.7	33.9	36.0 *	N/A	38.1 *	N/A	N/A	120.2% *

I-PRF—injectable platelet-rich fibrin; AC—arthrocentesis; N/A—not applicable; * *p* < 0.05.

**Table 9:** TMD indices in the study and control groups.

First Author, Publication Year; TMD Index	Patient Group	Initial Value	After 7–10 Days	After 1 Month	After 3 Months	After 6 Months	Effect after 3 Months (the Lower the Better)
Ghoneim, 2022 [53]	AC + I-PRF	20.0	0.0 *	N/A	0.0 *	0.0 *	0.0% *
Clicking score	AC	20.0	12.0 *	N/A	6.0 *	9.0 *	30.0% *
Karadayi, 2021 [55]	AC + I-PRF	15.7	7.7	3.4 *	2.1 *	N/A	13.4% *
Helkimo index	AC	15.7	11.8 *	8.4 *	6.4 *	N/A	40.8% *

TMD—temporomandibular disorder; I-PRF—injectable platelet-rich fibrin; AC—arthrocentesis; N/A—not applicable; * *p* < 0.05.

## Data Availability

All collected data are presented in the body of this article in text, tables, and figures. The review protocol has not been previously published.

## References

[1] Marcelino V., De Rovere S., Paço M., Gonçalves M., Marcelino S., Guimarães A.S., Pinho T. (2023). Masticatory Function in Individuals with Temporomandibular Disorders: A Systematic Review and Meta-Analysis. Life.

[2] Vieira L.S., Pestana P.R.M., Miranda J.P., Soares L.A., Silva F., Alcantara M.A., Oliveira V.C. (2023). The Efficacy of Manual Therapy Approaches on Pain, Maximum Mouth Opening and Disability in Temporomandibular Disorders: A Systematic Review of Randomised Controlled Trials. Life.

[3] Greenbaum T., Pitance L., Kedem R., Emodi-Perlman A. (2022). The Mouth-opening Muscular Performance in Adults with and without Temporomandibular Disorders: A Systematic Review. J. Oral Rehabil..

[4] Sójka A., Huber J., Hędzelek W., Wiertel-Krawczuk A., Szymankiewicz-Szukała A., Seraszek-Jaros A., Kulczyk A., Wincek A., Sobieska M. (2018). Relations between the Results of Complex Clinical and Neurophysiological Examinations in Patients with Temporomandibular Disorders Symptoms. CRANIO®.

[5] Chęciński M., Sikora M., Chęcińska K., Nowak Z., Chlubek D. (2022). The Administration of Hyaluronic Acid into the Temporomandibular Joints’ Cavities Increases the Mandible’s Mobility: A Systematic Review and Meta-Analysis. J. Clin. Med..

[6] Tournavitis A., Sandris E., Theocharidou A., Slini T., Kokoti M., Koidis P., Tortopidis D. (2022). Effectiveness of Conservative Therapeutic Modalities for Temporomandibular Disorders-Related Pain: A Systematic Review. Acta Odontol. Scand..

[7] Derwich M., Górski B., Amm E., Pawłowska E. (2023). Oral Glucosamine in the Treatment of Temporomandibular Joint Osteoarthritis: A Systematic Review. Int. J. Mol. Sci..

[8] Turosz N., Chęcińska K., Chęciński M., Kamińska M., Nowak Z., Sikora M., Chlubek D. (2022). A Scoping Review of the Use of Pioglitazone in the Treatment of Temporo-Mandibular Joint Arthritis. Int. J. Environ. Res. Public Health.

[9] Müggenborg F., de Castro Carletti E.M., Dennett L., de Oliveira-Souza A.I.S., Mohamad N., Licht G., von Piekartz H., Armijo-Olivo S. (2023). Effectiveness of Manual Trigger Point Therapy in Patients with Myofascial Trigger Points in the Orofacial Region—A Systematic Review. Life.

[10] Brighenti N., Battaglino A., Sinatti P., Abuín-Porras V., Sánchez Romero E.A., Pedersini P., Villafañe J.H. (2023). Effects of an Interdisciplinary Approach in the Management of Temporomandibular Disorders: A Scoping Review. Int. J. Environ. Res. Public Health.

[11] Nitecka-Buchta A., Walczynska-Dragon K., Batko-Kapustecka J., Wieckiewicz M. (2018). Comparison between Collagen and Lidocaine Intramuscular Injections in Terms of Their Efficiency in Decreasing Myofascial Pain within Masseter Muscles: A Randomized, Single-Blind Controlled Trial. Pain Res. Manag..

[12] Nowak Z., Chęciński M., Nitecka-Buchta A., Bulanda S., Ilczuk-Rypuła D., Postek-Stefańska L., Baron S. (2021). Intramuscular Injections and Dry Needling within Masticatory Muscles in Management of Myofascial Pain. Systematic Review of Clinical Trials. Int. J. Environ. Res. Public Health.

[13] Arribas-Pascual M., Hernández-Hernández S., Jiménez-Arranz C., Grande-Alonso M., Angulo-Díaz-Parreño S., La Touche R., Paris-Alemany A. (2023). Effects of Physiotherapy on Pain and Mouth Opening in Temporomandibular Disorders: An Umbrella and Mapping Systematic Review with Meta-Meta-Analysis. J. Clin. Med..

[14] Thorpe A.R.D.S., Haddad Y., Hsu J. (2023). A Systematic Review and Meta-Analysis of Randomized Controlled Trials Comparing Arthrocentesis with Conservative Management for Painful Temporomandibular Joint Disorder. Int. J. Oral Maxillofac. Surg..

[15] Chęciński M., Chęcińska K., Nowak Z., Sikora M., Chlubek D. (2022). Treatment of Mandibular Hypomobility by Injections into the Temporomandibular Joints: A Systematic Review of the Substances Used. J. Clin. Med..

[16] Bhattacharjee B., Bera R.N., Verma A., Soni R., Bhatnagar A. (2023). Efficacy of Arthrocentesis and Stabilization Splints in Treatment of Temporomandibular Joint Disc Displacement Disorder Without Reduction: A Systematic Review and Meta-Analysis. J. Maxillofac. Oral Surg..

[17] Ozawa M., Okaue M., Kaneko K., Hasegawa M., Matsunaga S., Matsumoto M., Hori M., Kudo I., Takagi M. (1996). Clinical Assessment of the Pumping Technique in Treating TMJ Arthrosis with Closed Lock. J. Nihon Univ. Sch. Dent..

[18] Torres-Rosas R., Marcela Castro-Gutiérrez M., Flores-Mejía L., Torres-Rosas E., Nieto-García R., Argueta-Figueroa L. (2023). Ozone for the Treatment of Temporomandibular Joint Disorders: A Systematic Review and Meta-Analysis. Med. Gas Res..

[19] Sit R.W.-S., Reeves K.D., Zhong C.C., Wong C.H.L., Wang B., Chung V.C., Wong S.Y., Rabago D. (2021). Efficacy of Hypertonic Dextrose Injection (Prolotherapy) in Temporomandibular Joint Dysfunction: A Systematic Review and Meta-Analysis. Sci. Rep..

[20] Zarate M.A., Frusso R.D., Reeves K.D., Cheng A.-L., Rabago D. (2020). Dextrose Prolotherapy Versus Lidocaine Injection for Temporomandibular Dysfunction: A Pragmatic Randomized Controlled Trial. J. Altern. Complement. Med..

[21] Gutiérrez I.Q., Sábado-Bundó H., Gay-Escoda C. (2021). Intraarticular Injections of Platelet Rich Plasma and Plasma Rich in Growth Factors with Arthrocenthesis or Arthroscopy in the Treatment of Temporomandibular Joint Disorders: A Systematic Review. J. Stomatol. Oral Maxillofac. Surg..

[22] Haigler M.C., Abdulrehman E., Siddappa S., Kishore R., Padilla M., Enciso R. (2018). Use of Platelet-Rich Plasma, Platelet-Rich Growth Factor with Arthrocentesis or Arthroscopy to Treat Temporomandibular Joint Osteoarthritis: Systematic Review with Meta-Analyses. J. Am. Dent. Assoc..

[23] Chęciński M., Chęcińska K., Turosz N., Kamińska M., Nowak Z., Sikora M., Chlubek D. (2022). Autologous Stem Cells Transplants in the Treatment of Temporomandibular Joints Disorders: A Systematic Review and Meta-Analysis of Clinical Trials. Cells.

[24] Sikora M., Sielski M., Chęciński M., Nowak Z., Czerwińska-Niezabitowska B., Chlubek D. (2022). Repeated Intra-Articular Administration of Platelet-Rich Plasma (PRP) in Temporomandibular Disorders: A Clinical Case Series. J. Clin. Med..

[25] Giacomello M., Giacomello A., Mortellaro C., Gallesio G., Mozzati M. (2015). Temporomandibular Joint Disorders Treated with Articular Injection: The Effectiveness of Plasma Rich in Growth Factors-Endoret. J. Craniofac. Surg..

[26] Albilia J., Herrera-Vizcaíno C., Weisleder H., Choukroun J., Ghanaati S. (2020). Liquid Platelet-Rich Fibrin Injections as a Treatment Adjunct for Painful Temporomandibular Joints: Preliminary Results. Cranio J. Craniomandib. Pract..

[27] Derwich M., Mitus-Kenig M., Pawlowska E. (2021). Mechanisms of Action and Efficacy of Hyaluronic Acid, Corticosteroids and Platelet-Rich Plasma in the Treatment of Temporomandibular Joint Osteoarthritis—A Systematic Review. Int. J. Mol. Sci..

[28] Kałużyński K., Trybek G., Smektała T., Masiuk M., Myśliwiec L., Sporniak-Tutak K. (2016). Effect of Methylprednisolone, Hyaluronic Acid and Pioglitazone on Histological Remodeling of Temporomandibular Joint Cartilage in Rabbits Affected by Drug-Induced Osteoarthritis. Postep. Hig. Med. Dosw. Online.

[29] Sikora M., Chęciński M., Nowak Z., Chlubek D. (2021). Variants and Modifications of the Retroauricular Approach Using in Temporomandibular Joint Surgery: A Systematic Review. J. Clin. Med..

[30] Nogueira E.F.C., Lemos C.A.A., Vasconcellos R.J.H., Moraes S.L.D., Vasconcelos B.C.E., Pellizzer E.P. (2021). Does Arthroscopy Cause More Complications than Arthrocentesis in Patients with Internal Temporomandibular Joint Disorders? Systematic Review and Meta-Analysis. Br. J. Oral Maxillofac. Surg..

[31] Matheus H.R., Özdemir Ş.D., Guastaldi F.P.S. (2022). Stem Cell-Based Therapies for Temporomandibular Joint Osteoarthritis and Regeneration of Cartilage/Osteochondral Defects: A Systematic Review of Preclinical Experiments. Osteoarthr. Cartil..

[32] Pagotto L.E.C., de Santana Santos T., Pastore G.P. (2021). The Efficacy of Mesenchymal Stem Cells in Regenerating Structures Associated with the Temporomandibular Joint: A Systematic Review. Arch. Oral Biol..

[33] Minervini G., Del Mondo D., Russo D., Cervino G., D’Amico C., Fiorillo L. (2022). Stem Cells in Temporomandibular Joint Engineering: State of Art and Future Persectives. J. Craniofac. Surg..

[34] Bhalla M., El-Housseini Z., Asaria R. (2022). Corrigendum to “Blindness Associated with Platelet-Rich Plasma Temporomandibular Joint Injections” [Br. J. Oral Maxillofac. Surg. 58(9) (2020) 1197–1199]. Br. J. Oral Maxillofac. Surg..

[35] Putthirangsiwong B., Vongsilpavattana V., Leelawongs S., Chanthanaphak E., Tunlayadechanont P., Chokthaweesak W. (2022). Superior Ophthalmic Vein Embolism Following Forehead Augmentation with Autologous Fat Injection. Aesthetic Plast. Surg..

[36] Szantyr A., Orski M., Marchewka I., Szuta M., Orska M., Zapała J. (2017). Ocular Complications Following Autologous Fat Injections into Facial Area: Case Report of a Recovery from Visual Loss After Ophthalmic Artery Occlusion and a Review of the Literature. Aesthetic Plast. Surg..

[37] Farshidfar N., Jafarpour D., Firoozi P., Sahmeddini S., Hamedani S., de Souza R.F., Tayebi L. (2022). The Application of Injectable Platelet-Rich Fibrin in Regenerative Dentistry: A Systematic Scoping Review of In Vitro and In Vivo Studies. Jpn. Dent. Sci. Rev..

[38] Page M.J., McKenzie J.E., Bossuyt P.M., Boutron I., Hoffmann T.C., Mulrow C.D., Shamseer L., Tetzlaff J.M., Akl E.A., Brennan S.E. (2021). The PRISMA 2020 Statement: An Updated Guideline for Reporting Systematic Reviews. BMJ..

[39] Tricco A.C., Lillie E., Zarin W., O’Brien K.K., Colquhoun H., Levac D., Moher D., Peters M.D.J., Horsley T., Weeks L. (2018). PRISMA Extension for Scoping Reviews (PRISMA-ScR): Checklist and Explanation. Ann. Intern. Med..

[40] Schiavenato M., Chu F. (2021). PICO: What It Is and What It Is Not. Nurse Educ. Pract..

[41] Ouzzani M., Hammady H., Fedorowicz Z., Elmagarmid A. (2016). Rayyan—A Web and Mobile App for Systematic Reviews. Syst. Rev..

[42] Więckowska B., Kubiak K.B., Jóźwiak P., Moryson W., Stawińska-Witoszyńska B. (2022). Cohen’s Kappa Coefficient as a Measure to Assess Classification Improvement Following the Addition of a New Marker to a Regression Model. Int. J. Environ. Res. Public. Health.

[43] Ooi K., Aihara M., Matsumura H., Matsuda S., Watanabe Y., Yuasa H., Matsuka Y. (2022). Therapy Outcome Measures in Temporomandibular Disorder: A Scoping Review. BMJ Open.

[44] Idáñez-Robles A.M., Obrero-Gaitán E., Lomas-Vega R., Osuna-Pérez M.C., Cortés-Pérez I., Zagalaz-Anula N. (2023). Exercise Therapy Improves Pain and Mouth Opening in Temporomandibular Disorders: A Systematic Review with Meta-Analysis. Clin. Rehabil..

[45] Keller S., Bocell F.D., Mangrum R., McLorg A., Logan D., Chen A.L., Steen A.I., Woods P., Weinberg J., Royce L. (2023). Patient-Reported Outcome Measures for Individuals with Temporomandibular Joint Disorders: A Systematic Review and Evaluation. Oral Surg. Oral Med. Oral Pathol. Oral Radiol..

[46] OCEBM Levels of Evidence—Centre for Evidence-Based Medicine (CEBM), University of Oxford. https://www.cebm.ox.ac.uk/resources/levels-of-evidence/ocebm-levels-of-evidence.

[47] Sterne J.A.C., Savović J., Page M.J., Elbers R.G., Blencowe N.S., Boutron I., Cates C.J., Cheng H.-Y., Corbett M.S., Eldridge S.M. (2019). RoB 2: A Revised Tool for Assessing Risk of Bias in Randomised Trials. BMJ.

[48] Baiomy A.A., Edrees M., Al-Ashmawy M. (2019). Versatility of Injectable Platelet Rich Fibrin versus Autologous Blood Injection in the Treatment of Chronic Recurrent Temporomandibular Joint Dislocation. Egypt. J. Oral Maxillofac. Surg..

[49] Vingender S., Dőri F., Schmidt P., Hermann P., Vaszilkó M.T. (2023). Evaluation of the Efficiency of Hyaluronic Acid, PRP and I-PRF Intra-Articular Injections in the Treatment of Internal Derangement of the Temporomandibular Joint: A Prospective Study. J. Cranio-Maxillofac. Surg..

[50] Da Silva Lima G.L., de Souza Carvalho A.A., de Toledo Lourenço A.H. (2022). Ácido Hialurônico Vs Fibrina Rica em Plaquetas: Qual Utilizar em Tratamentos de Osteoartrite Temporomandibular?. Anais da II Jornada Odontológica Online.

[51] González L.V., López J.P., Díaz-Báez D., Orjuela M.P., Chavez M. (2021). Clinical Outcomes of Operative Arthroscopy and Temporomandibular Medical Infiltration with Platelet-Rich Fibrin in Upper and Lower Articular Space. J. Cranio-Maxillofac. Surg. Off. Publ. Eur. Assoc. Cranio-Maxillofac. Surg..

[52] Manafikhi M., Ataya J., Heshmeh O. (2022). Evaluation of the Efficacy of Platelet Rich Fibrin (I-PRF) Intra-Articular Injections in the Management of Internal Derangements of Temporomandibular Joints—A Controlled Preliminary Prospective Clinical Study. BMC Musculoskelet. Disord..

[53] Ghoneim N.I., Mansour N.A., Elmaghraby S.A., Abdelsameaa S.E. (2022). Treatment of Temporomandibular Joint Disc Displacement Using Arthrocentesis Combined with Injectable Platelet Rich Fibrin versus Arthrocentesis Alone. J. Dent. Sci..

[54] Işık G., Kenç S., Özveri Koyuncu B., Günbay S., Günbay T. (2022). Injectable Platelet-Rich Fibrin as Treatment for Temporomandibular Joint Osteoarthritis: A Randomized Controlled Clinical Trial. J. Cranio-Maxillofac. Surg..

[55] Karadayi U., Gursoytrak B. (2021). Randomised Controlled Trial of Arthrocentesis with or without PRF for Internal Derangement of the TMJ. J. Cranio-Maxillofac. Surg..

[56] Torul D., Cezairli B., Kahveci K. (2021). The Efficacy of Intra-Articular Injectable Platelet-Rich Fibrin Application in the Management of Wilkes Stage III Temporomandibular Joint Internal Derangement. Int. J. Oral Maxillofac. Surg..

[57] Yuce E., Komerik N. (2020). Comparison of the Efficiacy of Intra-Articular Injection of Liquid Platelet-Rich Fibrin and Hyaluronic Acid After in Conjunction with Arthrocentesis for the Treatment of Internal Temporomandibular Joint Derangements. J. Craniofac. Surg..

[58] Muhammad K., Khaldoun A. (2022). Evaluation of the Combination Efficacy of Injectable Platelet Rich Fibrin (I-PRF) with Ultrasound as an Adjunctive Therapy in the Management of TMJ Pain (Clinical Study). Damascus Univ. J. Med. Sci..

[59] Chęciński M., Chęcińska K., Turosz N., Sikora M., Chlubek D. (2023). Intra-Articular Injections into the Inferior versus Superior Compartment of the Temporomandibular Joint: A Systematic Review and Meta-Analysis. J. Clin. Med..

[60] Gürsoytrak B. (2020). Evaluation of The Clinical Efficacy of Liquid Platelet Rich Fibrin Application in Painful Temporomandibular Joint Disorders. https://clinicaltrials.gov/ct2/show/NCT04317560.

[61] Smardz J. (2022). Assessment of the Effectiveness of Intra-Articular Injectable Platelet-Rich Fibrin (IPRF) Injections in the Management of Mild and Moderate Degeneration of the Temporomandibular Joints. https://clinicaltrials.gov/ct2/show/NCT05214924.

[62] Barath Z., Rasko Z. (2020). Technique for Achieving a Stable Position of the Condylar Process during Injection into the Temporomandibular Joint. Br. J. Oral Maxillofac. Surg..

[63] Champs B., Corre P., Hamel A., Laffite C.D., Le Goff B. (2019). US-Guided Temporomandibular Joint Injection: Validation of an in-Plane Longitudinal Approach. J. Stomatol. Oral Maxillofac. Surg..

[64] Matheson E.M., Fermo J.D., Blackwelder R.S. (2023). Temporomandibular Disorders: Rapid Evidence Review. Am. Fam. Physician.

[65] Sikora M., Czerwińska-Niezabitowska B., Chęciński M.A., Sielski M., Chlubek D. (2020). Short-Term Effects of Intra-Articular Hyaluronic Acid Administration in Patients with Temporomandibular Joint Disorders. J. Clin. Med..

[66] Torres D., Zaror C., Iturriaga V., Tobias A. (2020). Intra-Articular Corticosteroids for Treatment of Temporomandibular Joint Internal Disorders: Protocol for Systematic Review and Network Meta-Analysis. BMJ Open.

[67] Ferreira N., Masterson D., Lopes de Lima R., de Souza Moura B., Oliveira A.T., Kelly da Silva Fidalgo T., Carvalho A.C.P., DosSantos M.F., Grossmann E. (2018). Efficacy of Viscosupplementation with Hyaluronic Acid in Temporomandibular Disorders: A Systematic Review. J. Cranio-Maxillofac. Surg. Off. Publ. Eur. Assoc. Cranio-Maxillofac. Surg..

[68] Moldez M.A., Camones V.R., Ramos G.E., Padilla M., Enciso R. (2018). Effectiveness of Intra-Articular Injections of Sodium Hyaluronate or Corticosteroids for Intracapsular Temporomandibular Disorders: A Systematic Review and Meta-Analysis. J. Oral Facial Pain Headache.

